# Bis[2-(2-pyridyl­sulfan­yl)eth­yl]ammonium perchlorate

**DOI:** 10.1107/S1600536810032216

**Published:** 2010-08-18

**Authors:** Guo-Qing Wang, Cong-Hui Ma, Xiao-Feng Li, Wen-Ge Li, Seik Weng Ng

**Affiliations:** a Shanghai Sunvea Chemical Materials Science and Technology Co. Ltd, Shanghai 201611, People’s Republic of China; b Qingdao University of Science and Technology, Qingdao 266042, People’s Republic of China; c Institute of Marine Materials Science and Engineering, Shanghai Maritime University, Shanghai 201306, People’s Republic of China; d Department of Chemistry, University of Malaya, 50603 Kuala Lumpur, Malaysia

## Abstract

The cation and anion of the title salt, C_14_H_18_N_3_S_2_^+^·ClO_4_^−^, lie on a twofold rotation axis. The cation is a W-shaped entity with the aromatic rings at the ends; the ammonium NH_2_^+^ group is a hydrogen-bond donor to the pyridyl N atoms. The perchlorate ion has one O atom disordered over two sites in a 0.50:0.50 ratio.

## Related literature

For the structure of tris­[2-(2-pyridyl­sulfan­yl)eth­yl]ammonium perchlorate, see: An *et al.* (2010 ▶).
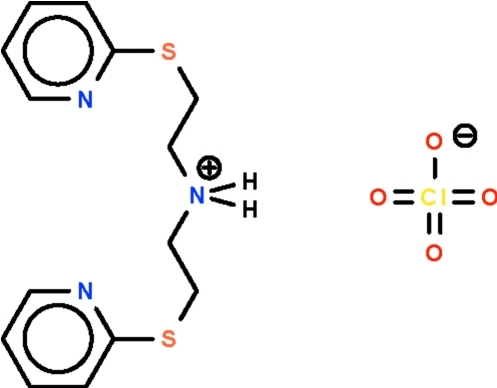


## Experimental

### 

#### Crystal data

C_14_H_18_N_3_S_2_^+^·ClO_4_^−^
*M* *_r_* = 391.88
Monoclinic, 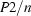

*a* = 8.1265 (6) Å
*b* = 9.2291 (7) Å
*c* = 11.9872 (9) Å
β = 97.534 (7)°
*V* = 891.28 (12) Å^3^
*Z* = 2
Cu *K*α radiation
μ = 4.31 mm^−1^
*T* = 293 K
0.15 × 0.15 × 0.10 mm

#### Data collection

Oxford Xcalibur Sapphire-3 diffractometer
Absorption correction: multi-scan (*CrysAlis RED*; Oxford Diffraction, 2009 ▶) *T*_min_ = 0.345, *T*_max_ = 1.000
3124 measured reflections
1736 independent reflections
1427 reflections with *I* > 2σ(*I*)
*R*_int_ = 0.020

#### Refinement

*R*[*F* ^2^ > 2σ(*F* ^2^)] = 0.071
*wR*(*F* ^2^) = 0.214
*S* = 1.11
1736 reflections
120 parameters
9 restraints
H-atom parameters constrained
Δρ_max_ = 0.41 e Å^−3^
Δρ_min_ = −0.66 e Å^−3^

### 

Data collection: *CrysAlis PRO* (Oxford Diffraction, 2009 ▶); cell refinement: *CrysAlis PRO*; data reduction: *CrysAlis PRO*; program(s) used to solve structure: *SHELXS97* (Sheldrick, 2008 ▶); program(s) used to refine structure: *SHELXL97* (Sheldrick, 2008 ▶); molecular graphics: *X-SEED* (Barbour, 2001 ▶); software used to prepare material for publication: *publCIF* (Westrip, 2010 ▶).

## Supplementary Material

Crystal structure: contains datablocks global, I. DOI: 10.1107/S1600536810032216/bh2303sup1.cif

Structure factors: contains datablocks I. DOI: 10.1107/S1600536810032216/bh2303Isup2.hkl

Additional supplementary materials:  crystallographic information; 3D view; checkCIF report

## Figures and Tables

**Table 1:** Hydrogen-bond geometry (Å, °)

*D*—H⋯*A*	*D*—H	H⋯*A*	*D*⋯*A*	*D*—H⋯*A*
N2—H2⋯N1	0.86	2.11	2.832 (5)	141

**Fig. 1.**
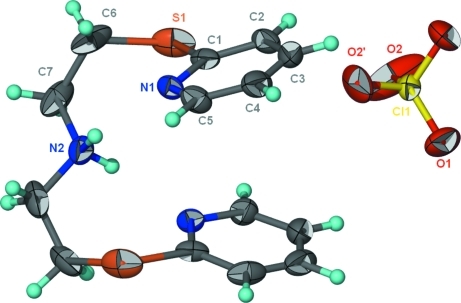
Thermal ellipsoid plot (Barbour, 2001) of [C14H18N3S2]+[ClO4]- at the 50% probability level; hydrogen atoms are drawn as spheres of arbitrary radius. For the cation, the unlabeled atoms are related to the labeled ones by symmetry 1/2-x, y, 3/2-z.

## supplementary crystallographic information

### Comment

We are engaged in the study of metal complexes of di- and tri-(pyridylsulfanyl)alkylamines as such compounds owing to their flexible nature. We reported earlier the synthesis of tris[2-(2-pyridylsulfanyl)ethyl]amine, whose reaction with copper perchlorate gave instead tris[2-(2-pyridylsulfanyl)ethyl]ammonium perchlorate (An *et al.*, 2010). The two-legged bis[2-(2-pyridylsulfanyl)ethyl]amine, in the present study, reacted with copper perchlorate to afford the corresponding ammonium perchlorate (Scheme and Fig. 1).

### Experimental

Bis(2-chloroethyl)ammonium hydrochloride (8.92 g, 0.05 mol) in ethanol (100 ml) was added to a solution (353 K) of 2-mercaptopyridine (12.23 g, 0.11 mol) and potassium hydroxide (6.17 g, 0.11 mol) in ethanol (200 ml). The mixture was heated at 353 K for 8 h. The solvent was removed to yield a yellow oil; this was column chromatographed with ethly acetate/petroleum ether (3/5 *v*/*v*) as eluent; yield 65%. ^1^H NMR (CDCl_3_, 400 MHz, p.p.m.): 3.316–3.349 (*t*, 4H), 2.959–2.992 (*t*, 4H), 6.924–6.960 (*m*, 2H), 7.154–7.173 (*m*, 2H), 7.416–7.459 (*m*, 2H), 8.382–8.393 (*m*, 2H).

The title salt was obtained from the reaction of bis[2-(2-pyridylsulfanyl)ethyl]amine (0.5 mmol, 0.146 g) and copper perchlorate (0.5 mmol, 0.132 g) in ethanol. Colorless crystals were separated from the blue solution after three days. CH&N elemental analysis, calculated for C_14_H_18_O_4_N_3_S_2_Cl: C 42.91, H 4.63, N 10.72%; Found: C 42.73, H 4.35, N 11.02%.

### Refinement

Carbon-bound H-atoms were placed in calculated positions (C—H 0.93 to 0.97 Å) and were included in the refinement in the riding model approximation, with *U*_iso_(H) set to 1.2 times *U*_eq_(C). The ammonium H-atom was similarly positioned [N—H 0.86 Å] and its temperature factor tied by a factor of 1.2.

The perchlorate ion is disordered about the twofold rotation axis with respect to one O atom; two O atoms were assigned half-occupancy. The Cl—O distances were restrained to within 0.005 Å of each other, as were the O···O separations.

### Crystal data

**Table:**

C_14_H_18_N_3_S_2_^+^·ClO_4_^−^	*F*(000) = 408
*M**_r_* = 391.88	*D*_x_ = 1.460 Mg m^−^^3^
Monoclinic, *P*2/*n*	Cu *K*α radiation, λ = 1.54184 Å
Hall symbol: -P 2yac	Cell parameters from 1783 reflections
*a* = 8.1265 (6) Å	θ = 3.7–72.5°
*b* = 9.2291 (7) Å	µ = 4.31 mm^−^^1^
*c* = 11.9872 (9) Å	*T* = 293 K
β = 97.534 (7)°	Prism, yellow
*V* = 891.28 (12) Å^3^	0.15 × 0.15 × 0.10 mm
*Z* = 2	

### Data collection

**Table:**

Oxford Xcalibur Sapphire-3 diffractometer	1736 independent reflections
Radiation source: fine-focus sealed tube	1427 reflections with *I* > 2σ(*I*)
graphite	*R*_int_ = 0.020
Detector resolution: 16.0855 pixels mm^-1^	θ_max_ = 72.7°, θ_min_ = 4.8°
ω scans	*h* = −6→9
Absorption correction: multi-scan (*CrysAlis RED*; Oxford Diffraction, 2009)	*k* = −11→10
*T*_min_ = 0.345, *T*_max_ = 1.000	*l* = −14→14
3124 measured reflections	

### Refinement

**Table:**

Refinement on *F*^2^	Secondary atom site location: difference Fourier map
Least-squares matrix: full	Hydrogen site location: inferred from neighbouring sites
*R*[*F*^2^ > 2σ(*F*^2^)] = 0.071	H-atom parameters constrained
*wR*(*F*^2^) = 0.214	*w* = 1/[σ^2^(*F*_o_^2^) + (0.1333*P*)^2^ + 0.2886*P*] where *P* = (*F*_o_^2^ + 2*F*_c_^2^)/3
*S* = 1.11	(Δ/σ)_max_ < 0.001
1736 reflections	Δρ_max_ = 0.41 e Å^−^^3^
120 parameters	Δρ_min_ = −0.66 e Å^−^^3^
9 restraints	Extinction correction: *SHELXL97* (Sheldrick, 2008), Fc^*^=kFc[1+0.001xFc^2^λ^3^/sin(2θ)]^-1/4^
0 constraints	Extinction coefficient: 0.077 (7)
Primary atom site location: structure-invariant direct methods	

### Fractional atomic coordinates and isotropic or equivalent isotropic displacement parameters (Å^2^)

**Table:**

	*x*	*y*	*z*	*U*_iso_*/*U*_eq_	Occ. (<1)
Cl1	0.7500	−0.07951 (11)	0.7500	0.0585 (5)	
S1	0.15849 (15)	0.6905 (2)	0.51373 (9)	0.1143 (7)	
O1	0.7006 (6)	−0.1628 (4)	0.8355 (3)	0.1259 (15)	
O2	0.6180 (11)	−0.001 (2)	0.6944 (18)	0.231 (14)	0.50
O2'	0.8847 (16)	0.0079 (15)	0.7855 (10)	0.184 (11)	0.50
N1	0.4045 (4)	0.6093 (3)	0.6688 (2)	0.0637 (8)	
N2	0.2500	0.8558 (5)	0.7500	0.0789 (13)	
H2	0.3345	0.8007	0.7463	0.095*	
C1	0.2997 (4)	0.5641 (5)	0.5824 (3)	0.0703 (10)	
C2	0.2947 (7)	0.4216 (6)	0.5433 (4)	0.0947 (16)	
H2A	0.2197	0.3938	0.4817	0.114*	
C3	0.4023 (10)	0.3248 (6)	0.5977 (6)	0.108 (2)	
H3	0.4022	0.2290	0.5736	0.130*	
C4	0.5080 (8)	0.3672 (5)	0.6857 (5)	0.1001 (16)	
H4	0.5815	0.3016	0.7244	0.120*	
C5	0.5067 (6)	0.5111 (5)	0.7187 (3)	0.0844 (12)	
H5	0.5823	0.5401	0.7796	0.101*	
C6	0.2658 (7)	0.8617 (6)	0.5449 (5)	0.115 (2)	
H6A	0.3843	0.8432	0.5566	0.137*	
H6B	0.2433	0.9242	0.4796	0.137*	
C7	0.2201 (7)	0.9415 (5)	0.6454 (6)	0.114 (2)	
H7A	0.2841	1.0304	0.6551	0.137*	
H7B	0.1036	0.9677	0.6318	0.137*	

### Atomic displacement parameters (Å^2^)

**Table:**

	*U*^11^	*U*^22^	*U*^33^	*U*^12^	*U*^13^	*U*^23^
Cl1	0.0546 (7)	0.0504 (6)	0.0705 (7)	0.000	0.0085 (4)	0.000
S1	0.0748 (8)	0.1960 (17)	0.0662 (7)	0.0179 (8)	−0.0137 (5)	0.0078 (7)
O1	0.178 (4)	0.106 (2)	0.101 (3)	0.005 (3)	0.046 (3)	0.039 (2)
O2	0.107 (12)	0.31 (3)	0.29 (2)	0.088 (14)	0.074 (13)	0.21 (2)
O2'	0.185 (18)	0.188 (15)	0.198 (14)	−0.139 (14)	0.098 (13)	−0.118 (12)
N1	0.0638 (17)	0.0756 (18)	0.0492 (14)	−0.0023 (13)	−0.0020 (12)	−0.0074 (12)
N2	0.077 (3)	0.061 (2)	0.103 (4)	0.000	0.030 (3)	0.000
C1	0.0612 (19)	0.104 (3)	0.0463 (16)	−0.0205 (18)	0.0108 (13)	−0.0068 (17)
C2	0.100 (3)	0.119 (4)	0.069 (2)	−0.050 (3)	0.025 (2)	−0.036 (3)
C3	0.155 (5)	0.076 (3)	0.108 (4)	−0.032 (3)	0.071 (4)	−0.020 (3)
C4	0.143 (5)	0.076 (3)	0.089 (3)	0.022 (3)	0.043 (3)	0.008 (2)
C5	0.098 (3)	0.090 (3)	0.062 (2)	0.016 (2)	−0.0026 (19)	−0.005 (2)
C6	0.100 (3)	0.137 (4)	0.113 (4)	0.027 (3)	0.037 (3)	0.071 (4)
C7	0.095 (3)	0.082 (3)	0.174 (5)	0.024 (3)	0.052 (4)	0.054 (3)

### Geometric parameters (Å, °)

**Table:**

Cl1—O2'	1.381 (4)	C2—C3	1.357 (8)
Cl1—O1^i^	1.383 (3)	C2—H2A	0.9300
Cl1—O1	1.383 (3)	C3—C4	1.329 (10)
Cl1—O2	1.391 (4)	C3—H3	0.9300
S1—C1	1.763 (4)	C4—C5	1.387 (7)
S1—C6	1.820 (7)	C4—H4	0.9300
N1—C1	1.319 (4)	C5—H5	0.9300
N1—C5	1.319 (5)	C6—C7	1.500 (9)
N2—C7	1.475 (6)	C6—H6A	0.9700
N2—C7^ii^	1.475 (6)	C6—H6B	0.9700
N2—H2	0.8600	C7—H7A	0.9700
C1—C2	1.395 (7)	C7—H7B	0.9700
			
O2'—Cl1—O1^i^	104.9 (8)	C2—C3—H3	120.1
O2'—Cl1—O1	113.1 (4)	C3—C4—C5	118.7 (5)
O1^i^—Cl1—O1	112.4 (3)	C3—C4—H4	120.6
O2'—Cl1—O2	111.9 (4)	C5—C4—H4	120.6
O1^i^—Cl1—O2	102.5 (12)	N1—C5—C4	123.8 (5)
O1—Cl1—O2	111.4 (4)	N1—C5—H5	118.1
O2^i^—Cl1—O2	117 (2)	C4—C5—H5	118.1
C1—S1—C6	102.3 (2)	C7—C6—S1	115.4 (3)
C1—N1—C5	116.3 (4)	C7—C6—H6A	108.4
C7—N2—C7^ii^	115.2 (6)	S1—C6—H6A	108.4
C7—N2—H2	108.5	C7—C6—H6B	108.4
C7^ii^—N2—H2	108.5	S1—C6—H6B	108.4
N1—C1—C2	123.3 (4)	H6A—C6—H6B	107.5
N1—C1—S1	118.1 (3)	N2—C7—C6	112.9 (4)
C2—C1—S1	118.6 (3)	N2—C7—H7A	109.0
C3—C2—C1	118.1 (4)	C6—C7—H7A	109.0
C3—C2—H2A	121.0	N2—C7—H7B	109.0
C1—C2—H2A	121.0	C6—C7—H7B	109.0
C4—C3—C2	119.8 (5)	H7A—C7—H7B	107.8
C4—C3—H3	120.1		
			
C5—N1—C1—C2	−0.5 (5)	C2—C3—C4—C5	−0.9 (8)
C5—N1—C1—S1	179.2 (3)	C1—N1—C5—C4	−0.3 (6)
C6—S1—C1—N1	25.3 (3)	C3—C4—C5—N1	1.0 (8)
C6—S1—C1—C2	−155.0 (3)	C1—S1—C6—C7	−94.7 (4)
N1—C1—C2—C3	0.6 (6)	C7^ii^—N2—C7—C6	154.1 (5)
S1—C1—C2—C3	−179.1 (3)	S1—C6—C7—N2	58.4 (5)
C1—C2—C3—C4	0.1 (7)		

Symmetry codes: (i) −*x*+3/2, *y*, −*z*+3/2; (ii) −*x*+1/2, *y*, −*z*+3/2.

### Hydrogen-bond geometry (Å, °)

**Table:**

*D*—H···*A*	*D*—H	H···*A*	*D*···*A*	*D*—H···*A*
N2—H2···N1	0.86	2.11	2.832 (5)	141
